# Purification and characterization of inorganic pyrophosphatase for in vitro RNA transcription

**DOI:** 10.1139/bcb-2022-0118

**Published:** 2022-08-04

**Authors:** Scott Tersteeg, Tyler Mrozowich, Amy Henrickson, Borries Demeler, Trushar R. Patel

**Affiliations:** a Department of Chemistry and Biochemistry, Alberta RNA Research and Training Institute, University of Lethbridge, 4401 University Drive, Lethbridge, AB T1K 3M4, Canada;; b Department of Chemistry and Biochemistry, University of Montana, Missoula, MT 59812, USA;; c Li Ka Shing Institute of Virology, University of Alberta, Edmonton, AB T6G 2E1, Canada;; d Department of Microbiology, Immunology & Infectious Diseases, Cumming School of Medicine, University of Calgary, Calgary, AB T2N 4N1, Canada

**Keywords:** inorganic pyrophosphatase, RNA in vitro transcription, iPPase, analytical ultracentrifuge, small-angle X-ray scattering, pyrophosphatase inorganique, transcription in vitro de l’ARN, iPPase, ultracentrifugeuse analytique, diffusion des rayons X aux petits angles

## Abstract

Inorganic pyrophosphatase (iPPase) is an enzyme that cleaves pyrophosphate into two phosphate molecules. This enzyme is an essential component of in vitro transcription (IVT) reactions for RNA preparation as it prevents pyrophosphate from precipitating with magnesium, ultimately increasing the rate of the IVT reaction. Large-scale RNA production is often required for biochemical and biophysical characterization studies of RNA, therefore requiring large amounts of IVT reagents. Commercially purchased iPPase is often the most expensive component of any IVT reaction. In this paper, we demonstrate that iPPase can be produced in large quantities and high quality using a reasonably generic laboratory facility and that laboratory-purified iPPase is as effective as commercially available iPPase. Furthermore, using size exclusion chromatography coupled with multi-angle light scattering and dynamic light scattering, analytical ultracentrifugation, and small-angle X-ray scattering, we demonstrate that yeast iPPase can form tetramers and hexamers in solution as well as the enzymatically active dimer. Our work provides a robust protocol for laboratories involved with RNA in vitro transcription to efficiently produce active iPPase, significantly reducing the financial strain of large-scale RNA production.

## Résumé

La pyrophosphatase inorganique (iPPase) est une enzyme qui clive le pyrophosphate en deux molécules de phosphate. Cette enzyme est un composant essentiel des réactions de transcription in vitro (TIV) pour la préparation d’ARN, car elle empêche le pyrophosphate de précipiter avec le magnésium, ce qui augmente finalement la vitesse de la réaction de TIV. La production d’ARN à grande échelle est souvent requise pour les études de caractérisation biochimique et biophysique de l’ARN, ce qui nécessite de grandes quantités de réactifs pour la TIV. L’iPPase achetée dans le commerce est souvent le composant le plus cher de toute réaction de TIV. Dans cet article, les auteurs démontrent que l’iPPase peut être produite en grandes quantités avec une haute qualité en utilisant une installation de laboratoire raisonnablement générique, et que l’iPPase purifiée en laboratoire est aussi efficace que l’iPPase disponible dans le commerce. De plus, en utilisant la chromatographie d’exclusion stérique couplée à la diffusion multiangle de la lumière et à la diffusion dynamique de la lumière, l’ultracentrifugation analytique et la diffusion de rayons X aux petits angles, ils démontrent que l’iPPase de levure peut former des tétramères et des hexamères en solution, ainsi que le dimère enzymatiquement actif. Ce travail offre un protocole robuste pour les laboratoires engagés dans la transcription in vitro d’ARN afin de produire efficacement l’iPPase active, réduisant ainsi de manière significative les contraintes financières de la production d’ARN à grande échelle. [Traduit par la Rédaction]

## Introduction

Inorganic pyrophosphatase (iPPase; EC 3.6.1.1) is primarily characterized by its function to catalyze the hydrolysis of pyrophosphate into two monophosphates (Harold 1966), as presented in eq. 1. Three different families of iPPase are found in cells. First, families 1 and 2 are free-floating in the cytosol. They are free-floating in the cytosol and help remove pyrophosphates from the cellular environment. Though families 1 and 2 perform similar functions, they are evolutionarily unrelated proteins (Hughes et al. 2012). Family 1 is found in all kingdoms of life, has a greater affinity to Mg^2+^ ions than other cofactors, can hydrolyze approximately 200 molecules per second, and only has one domain per subunit (Kajander et al. 2013). Family 2 is predominantly found in bacterial species, has two domains per subunit, has a greater affinity to Mn^2+^ ions than other cofactors, and can hydrolyze approximately 2000 pyrophosphates per second. Family 3 or membrane-integral pyrophosphatases are membrane-bound in the mitochondria (Holmes et al. 2019). The membrane-bound iPPase functions primarily as an ion pump, coupling the hydrolysis to move H^+^ or Na^+^ across the membrane. While membrane-bound iPPases are essential, family 1 and 2 iPPases are more abundant in cells. Family 1 iPPase is the most studied iPPase and is the primary focus of our work.


(1) $${\text{P}}_{2}{\text{O}}_{7}^{4-}\left({\text{PP}}_{\text{i}}\right)+{\text{H}}_{2}\text{O}\overset{\;}{\overset{\text{iPPase }+{\text{Mg}}^{2+}}{\to }}2{\text{HPO}}_{4}^{2-}\left({\text{P}}_{\text{i}}\right)$$

Family 1 iPPase is present in numerous organisms and displays variations in the structural arrangement. For example, the bacterial iPPase predominately exists as a hexamer, consisting of subunits of approximately 20 kDa (Harutyunyan et al. 1997). Yeast iPPase, however, forms a dimer in its active state with monomers of approximately 32 kDa (Heinrikson et al. 1973). Family 1 iPPase is an essential enzyme for anabolic pathways such as RNA and DNA biosynthesis, and its removal has shown lethality in fungal cells (Lundin et al. 1992).

IPPase is an essential component not only for cells to synthesize RNA but also for in vitro transcription (IVT) reactions. IVTs are expensive reactions that can produce large amounts of RNA, which is needed for almost all in vitro RNA biological experiments, including RNA structure–function characterization studies and the development of RNA-based therapies (Aguilar et al. 2022). IPPase drastically improves the yield of RNA through a two-fold mechanism (Arnold et al. 2001). First, iPPase removes excess pyrophosphate produced during an IVT reaction (eq. 1), which can build up, inhibiting the enzymatic reaction in which pyrophosphate is a by-product. Second, iPPase prevents magnesium pyrophosphate formation, whereas Mg^2+^ ions are essential cofactors of T7 RNA polymerase, and without them, the functionality of T7 is dramatically reduced (Akama et al. 2012). By hydrolyzing the pyrophosphate before forming magnesium pyrophosphate, iPPase ensures that magnesium ions stay in the solution, optimizing T7 polymerase activity. With RNA therapeutics becoming increasingly popular, cost-effective methods must be developed to relieve the financial burden of IVT reactions so that more labs can perform research on RNA-based systems.

This paper demonstrates that large-scale laboratory production of yeast iPPase is feasible, effective, and a viable alternative to commercially purchased iPPase. Additionally, by utilizing size exclusion chromatography coupled with multi-angle light scattering and dynamic light scattering (SEC–MALS–DLS) and analytical ultracentrifugation (AUC), we show that yeast iPPase forms higher order oligomeric tetramers and hexamers in solution as well as the commonly seen active dimer. Furthermore, we show the low-resolution structure of the iPPase tetramer in solution using small-angle X-ray scat tering (SAXS) and the high-resolution structure via computationally predicted docking (SUPCOMB). Overall, characterization of higher order solution structures of yeast iPPase can contribute to a greater understanding of the broad structural features of family 1 inorganic phosphatases.

## Materials and methods

### Overexpression and purification of yeast inorganic pyrophosphatase

Plasmid pET29b-IPP1-His was a gift from Sebastian Maerkl and Takuya Ueda (Addgene plasmid # 124137) for overexpression of yeast gene IPP1. Lemo21(DE3) *Escherichia coli* cells were transformed with the original plasmid via heat shock and plated onto LB-kanamycin plates.

Two 500 mL flasks of autoinduction media (Formedium^™^, Norfolk, UK) were induced with a single bacterial colony per flask and 0.1% volume of kanamycin (50 mg/mL) and 0.1% of chloramphenicol (50 mg/mL). Bacterial cells were incubated with shaking at 37 °C for 24 h, followed by a 20 °C incubation for 72 h. All subsequent steps were performed at 4 °C. The cell suspensions were harvested via centrifugation for 15 min at 5000 rcf. The total cell pellet (12.7 g) was resuspended in 30 mL of lysis buffer (50 mmol/L Tris, 100 mmol/L NaCl, 5% glycerol, pH 8.0) along with 1 mmol/L of PMSF, 5 mmol/L of BME, 300 mg of lysozyme, and 0.1 mg DNase crystals (Thermo Fisher Scientific, Saint-Laurent, QC, Canada) and 250 mg of deoxycholic acid. Cells were lysed via sonication and centrifuged at 30 000 rcf for 45 min. The supernatant was decanted and incubated with a 5 mL 50% slurry of Ni-NTA (Thermo Fisher Scientific, Saint-Laurent, QC, Canada) resin for 1 h with constant inversion. The resin mixture was added to a gravity flow column, and the lysate was flowed through twice. Twenty-five milliliters of wash buffer (50 mmol/L Tris, 100 mmol/L NaCl, 5% glycerol, 30 mmol/L imidazole, pH 8.0) was added to the column, and fractions were collected. Following the wash, 50 mL of elution buffer (50 mmol/L Tris, 100 mmol/L NaCl, 5% glycerol, and 500 mmol/L imidazole, pH 8.0) was added to the column, with fractions collected similar to before. Ten microliters of each collected fraction was mixed with 6× protein loading dye, heated to 95 °C for 5 min, and then loaded onto a 1.0 cm well PAGE casting plate (Bio-Rad Laboratories, Mississauga, ON, Canada). Finally, the samples were run on a 12% sodium dodecyl sulfate polyacrylamide gel electrophoresis (SDS-PAGE) for 80 min at 150 V, followed by Coomassie staining. Fractions with a pure protein band at the expected molecular weight were pooled and concentrated to 18 *μ*mol/L using an Amicon Ultra-15 Centrifugal Filter Unit (10 000 Da MWCO) (Millipore Canada Ltd., Etobi-coke, ON, Canada) in storage buffer (80 mmol/L Tris–HCl, Ph 7.5, 30 mmol/L MgCl_2_, 4 mmol/L spermidine, 20 mmol/L NaCl, 4 mmol/L DTT) and mixed 1:1 with 100% glycerol to a final concentration of 9 *μ*mol/L and stored at −20 °C in aliquots.

Affinity-purified iPPase was further purified using a Superdex 200 Increase 10/300 GL column (Global Life Science Solutions USA LLC, Marlborough, MA, USA) via an AKTA pure FPLC system (Global Life Science Solutions USA LLC, Marlborough, MA, USA) at 0.5 mL/min, using iPPase storage buffer. Fractions were collected, and each peak was run on a 12% SDS-PAGE as described above.

### SEC–MALS–DLS

Light scattering experiments were performed on a Dawn^®^(Wyatt Technology Corporation, Santa Barbara, CA, USA) MAS instrument with 18 detector angles utilizing a 658 nm laser. Additionally, an Optilab^®^ (Wyatt Technology Corporation, Santa Barbara, CA, USA) refractometer was fitted downstream to measure the solvent refractive index and provide the absolute concentration of iPPase. Upstream of both instruments was an SEC column (Superdex 200 increase 10/300 GL, Global Life Science Solutions, USA LLC, Marlborough, MA, USA) attached to an ÄKTA pure FPLC (SEC–MALS). Scattering experiments were performed at ambient temperature (25 °C) with 0.5 mL/min flow rate and iPPase storage buffer (80 mmol/L Tris–HCl, pH 7.5, 30 mmol/L MgCl_2_, 4 mmol/L spermidine, 20 mmol/L NaCl, 4 mmol/L DTT). The refractive index of the solvent was defined as 1.042 (20 °C), while the d*n*/d*c* (refractive index increment) value of 0.1850 mL/g was used for all iPPase measurements. A final concentration of 355 *μ*mol/L iPPase was injected in a total volume of 500 μL. Data were processed and analyzed using Astra v8.0.0.25 (Wyatt Technology Corporation, Santa Barbara, CA, USA). Absolute molecular weight (*M*_w_) was calculated using eq. 2, where *R*(*θ*) is Rayleigh’s ratio, *K* is the polymer constant, and *c* is the concentration of the solute.


(2) $$M_{\text{w}}=\frac{R(\theta )}{Kc}$$

Integrated DLS measurements were taken every 3 s to determine the diffusion coefficient (*D*_*τ*_) in the same scattering volume view by the MALS detector. To calculate the radius of hydration (*R*_H_), we used the Stokes–Einstein equation (eq. 3) (Pusey 1974), where *k*_B_ is the Boltzmann coefficient (1.380 × 10^−23^ kg·m^2^·s^−2^·K^−1^), *T* is the absolute temperature, and *η* is the viscosity of the medium (calculated to be 1.025). All values were calculated using ASTRA v8.0.0.25.


(3) $$D_{\tau }=\frac{k_{\text{B}}T}{6\pi \eta R_{\text{h}}}$$

### Analytical ultracentrifugation

AUC data for iPPase were collected via a Beckman Optima AUC centrifuge and an AN50-Ti rotor at 20 °C. The iPPase sample was loaded at 0.5 OD at 280 nm (6.8 μmol/L) and 0.5 OD at 220 nm (0.75 μmol/L) into Epon-2 channel centerpieces in iPPase AUC buffer (80 mmol/L Tris–HCl, pH 7.5, 30 mmol/L MgCl_2_, 20 mmol/L NaCl). Samples were centrifuged at 40 000 rpm, and scans were collected at 20 s intervals. We used the UltraScan-III package (11) to analyze sedimentation data via in-house supercomputer calculations. We analyzed the sedimentation velocity AUC data using two-dimensional spectrum analysis (Brookes et al. 2009) with simultaneous removal of time-invariant noise, meniscus, and bottom positions fitted, as described previously (Demeler 2010), followed by enhanced van Holde–Weischet analysis (Demeler and van Holde 2004). We estimated the buffer density and viscosity corrections with UltraScan (1.003 g/cm^3^ and 1.0367 cP, respectively). Hydrodynamic parameters were corrected to standard conditions (20 °C and water).

### Melting curve analysis

Melting curve analysis was performed on a Tycho (Nanotemper Technologies, Munich, Germany). Measurements were done in triplicate at multiple concentrations (50, 5, and 0.5 μmol/L) in Tycho standard capillaries (Nanotemper Technologies). Data analysis was performed via MO Affinity Analysis Software v2.1.3 (Nanotemper Technologies, Munich, Germany).

### Small-angle X-ray scattering

Sample X-ray scattering data collection (HPLC-SAXS) was performed at the B21 beamline at Diamond Light Source (Didcot, UK), as described previously (Meier et al. 2018). Our iPPase sample (50 μL) was injected into an in-line Agilent 1200 (Agilent Technologies, Stockport, UK) HPLC connected to a specialized flow cell at 450 μmol/L concentration and flowed through a buffer equilibrated Superdex 200 increase 3.2/300 GL (Global Life Science Solutions, USA LLC, Marlborough, MA, USA) size exclusion column at a flow rate of 0.160 mL/min. Each frame was exposed to the X-ray for 3 s. Peak regions were buffer subtracted and merged via CHROMIXS (Panjkovich and Svergun 2018). We analyzed the merged data using Guinier approximation to obtain the radius of gyration (*R*_g_) and study the homogeneity of samples (Guinier et al. 1956). Dimensionless Kratky analysis (Durand et al. 2010) was also utilized to determine whether the iPPase sample was folded, similar to previous examples (Nelson et al. 2021). Furthermore, we utilized pair-distance distribution (*P*(*r*)) analysis via GNOM (Svergun 1992) to provide the *R*_g_ and the maximum particle dimension (*D*_max_). Using information derived from the *P*(*r*) plot, models were generated using DAMMIN (Svergun 1999), without enforced symmetry, as described previously (Reuten et al. 2016). Finally, the previously generated models were averaged and filtered to obtain a single representative model through DAMAVER (Volkov and Svergun 2003). All SAXS data analysis packages are included in ATSAS Suite (version 3.0.5).

### High-resolution models with SAXS envelopes

We performed CLUSPRO (Kozakov et al. 2013, 2017; Xia et al. 2015; Vajda et al. 2017; Ignatov et al. 2018; Desta et al. 2020) docking analysis using our SAXS data as a constraint and PDB ID: 117e (Tuominen et al. 1998) (yeast iPPase) and generated 100 models. Using these 100 models, we then screened them against the raw scattering data using Crysol (Franke et al. 2017) to generate *χ*^2^ values based on the radius of gyration. Next, we visually inspected the top five results and overlaid them with our SAXS envelopes via SUPCOMB (Kozin and Svergun 2001).

### In vitro transcription to evaluate iPPase activity

RNA product is a 221 nucleotide length RNA transcript controlled by a T7 promoter and flanked on the 3^′^end by an XbaI digestion site cloned into a standard pUC-GW (Azenta Life Sciences, NJ, USA) plasmid. Three hundred microliters of 60 ng/μL plasmid DNA was linearized prior to IVT via an XbaI restriction digest site followed by heat inactivation (65 °C for 20 min). IVT ingredients (Table 1) were mixed in the order listed to determine the functionality of iPPase produced in-house versus the iPPase that was previously purchased from Sigma–Aldrich. T7 polymerase was produced and purified in-house, and Ribolock was purchased (Thermo Fisher Scientific, Saint-Laurent, QC). Samples were taken and quenched with an equal volume of 3 mol/L sodium acetate, pH 5.3. After 4 h at 37 °C, the reaction was quenched with an equal volume of 3 mol/L sodium acetate, pH 5.3. Ten microliters of each time point was mixed with RNA loading dye and heated to 95 °C before loading on a 1.0 cm well PAGE casting plate (Bio-Rad Laboratories, Mississauga, ON, Canada). Samples were run on a 7.5% urea-PAGE gel for 30 min at 300 V in 0.5× TBE buffer. The resulting gel was stained with SybrSafe (Thermo Fisher Scientific, Saint-Laurent, QC) and visualized.

## Results

### Purification of yeast inorganic pyrophosphatase

**Fig. 1.**
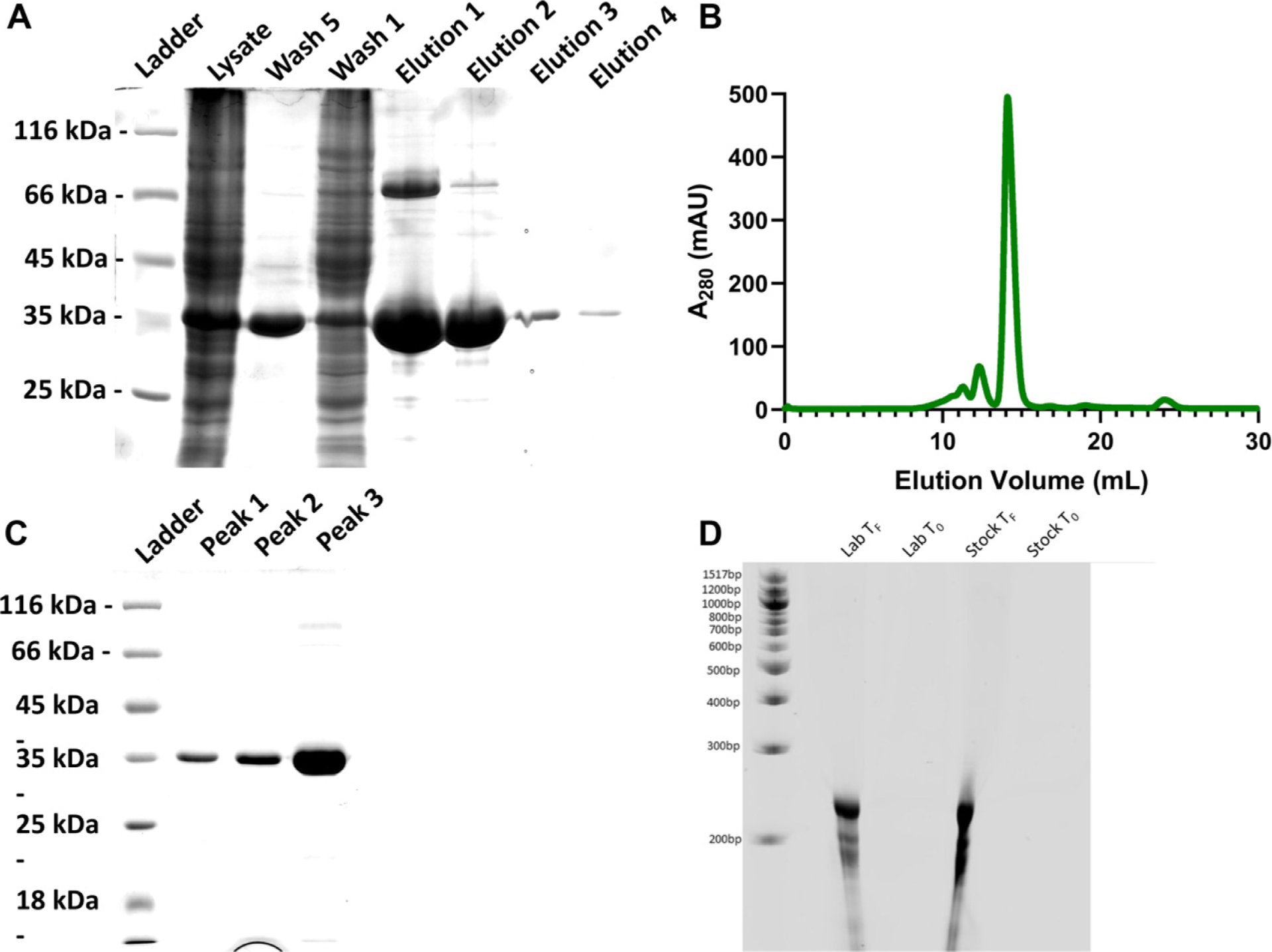
Purification of yeast iPPase. (A) SDS-PAGE after affinity purification via Ni-NTA. (B) Size exclusion chromatogram purification of pooled elutions from after affinity purification. (C) SDS-PAGE after size exclusion chromatography. (D) RNA preparation using in vitro transcription demonstrates that the laboratory-purified iPPase is active. *T*_F_ (time final) lanes show a similar amount of RNA being produced with laboratory-purified and the commercially purchased (stock) iPPase.

Yeast iPPase was expressed in Lemo21(DE3) *E. coli* cells, followed by the initial purification using affinity chromatography (Fig. 1A). The elution fractions show a high degree of expression of a band at the 35 kDa ladder size, which is consistent with monomeric iPPase (32 kDa). Elution 1 also contains a higher band at ~66 kDa and some minor contaminating species. Next, to ensure that the iPPase was homogeneous for downstream studies, affinity-purified preparation was subjected to SEC. The SEC chromatogram in Fig. 1B shows one predominant peak eluting at ~13.5–14.5 mL and two minor peaks eluting at ~11–12 and ~12–13 mL. These peak fractions were collected and further analyzed on an SDS-PAGE. As seen in Fig. 1C, all three peaks contain a distinct species of approximately 35 kDa in size, similar to the sequence molecular weight of iPPase (32 kDa) and consistent with affinity chromatography. Peak 3, similarly to affinity chromatography, shows an additional faint band above the monomeric iPPase species just above the 65 kDa ladder. Figure 1D shows the results of urea-PAGE analysis of in vitro transcribed RNA using commercially purchased iPPase (stock) and lab expressed and purified iPPase. Both *T*_F_ (time final) lanes show a similar amount of RNA being produced and a similar quality of RNA. Overall, we obtained approximately 250 mL of purified iPPase at 9 μmol/L concentration!

### Biophysical characterization of yeast iPPase

**Fig. 2.**
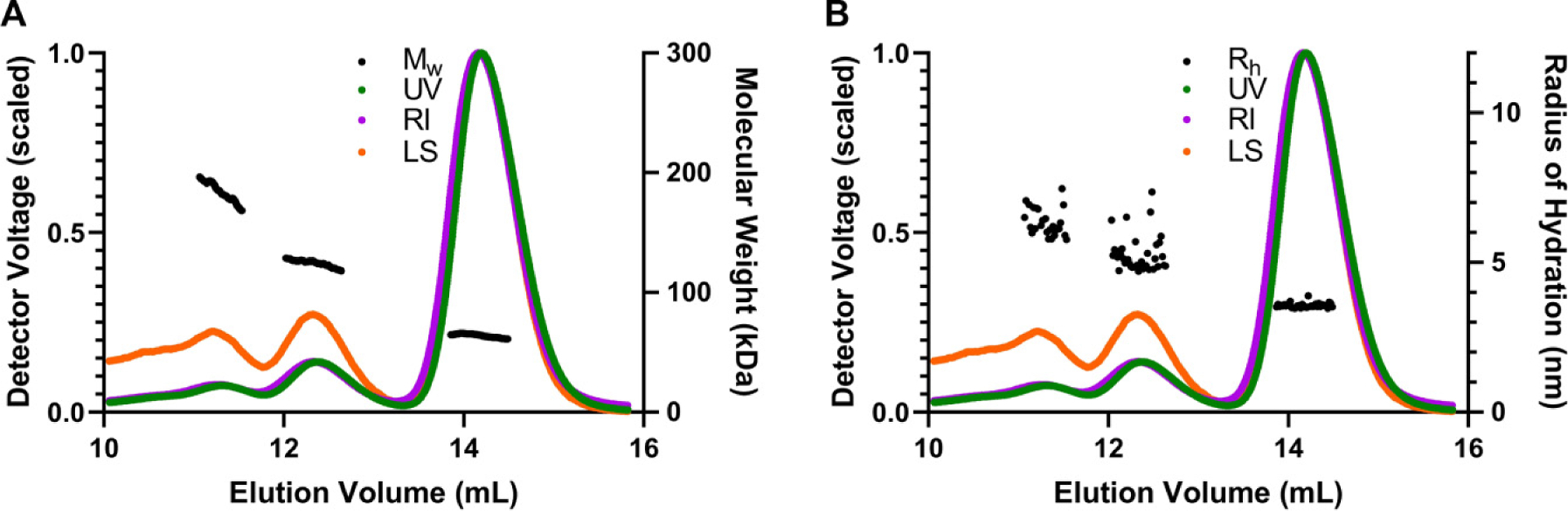
Light scattering analysis of purified iPPase. (A) Absolute molecular weight analysis of purified iPPase via multi-angle light scattering. The black line(s) represent the molecular weight across each solute peak. (B) Hydrodynamic radius analysis of purified iPPase via dynamic light scattering. Black point(s) represent the hydrodynamic radius across each solute peak. *R*_H_ is the hydrodynamic radius, RI is the refractive index, UV is 280 nm, and LS is the light scattering signal.

As we observed two additional peaks in Fig. 1C, we were curious to investigate the possibility of iPPase forming oligomeric conformations in solution. Therefore, the affinity-purified iPPase was subjected to SEC–MALS–DLS analysis. By coupling MALS and DLS with SEC, we obtained the absolute molecular weight for the three different peaks and the radius of hydration (*R*_H_), which are essential parameters for defining the size and shape of the proteins. The profile from SEC–MALS–DLS displayed similar trends as Fig. 1B. As presented in Fig. 2A, the peak eluting at ~13.5–15 mL has a molecular weight of 63.78 ± 1.54 kDa. Based on the sequence *M*_w_ of 32 kDa, this is consistent with yeast iPPase, which forms a functional dimer in solution. The peak eluting at ~12–13 mL has a molecular weight of 125.00 ± 3.20 kDa, which is the predicted weight of a tetramer. The final peak eluting from ~11–11.5 mL has a molecular weight of 186.90 5.45 kDa, the weight of predicted hexameric iPPase. Fig. 2B presents the *R*_H_ measurements derived from DLS with the peak eluting from ~13.5 to 15 mL, corresponding to dimeric iPPase, with an *R*_H_ of 3.56 ± 0.08 nm. The peak eluting at ~12–13 mL, corresponding to tetrameric iPPase assembly, has an *R*_H_ of 5.24 ± 0.14 nm. The hexamer peak eluted at ~11–11.5 with an *R*_H_ of 6.31 ± 0.16 nm. As expected, the *R*_H_ values of iPPase increase as the size of the molecular assembly increases. Furthermore, the mass fraction % ratio of 83.3%, 11.6%, and 5.1% was calculated for the dimer, tetramer, and hexamer, respectively. Additionally, the mass fraction % ratio was converted to a molar ratio of the dimer, tetramer, and hexamer, resulting in a 55:3:1 ratio.

**Fig. 3.**
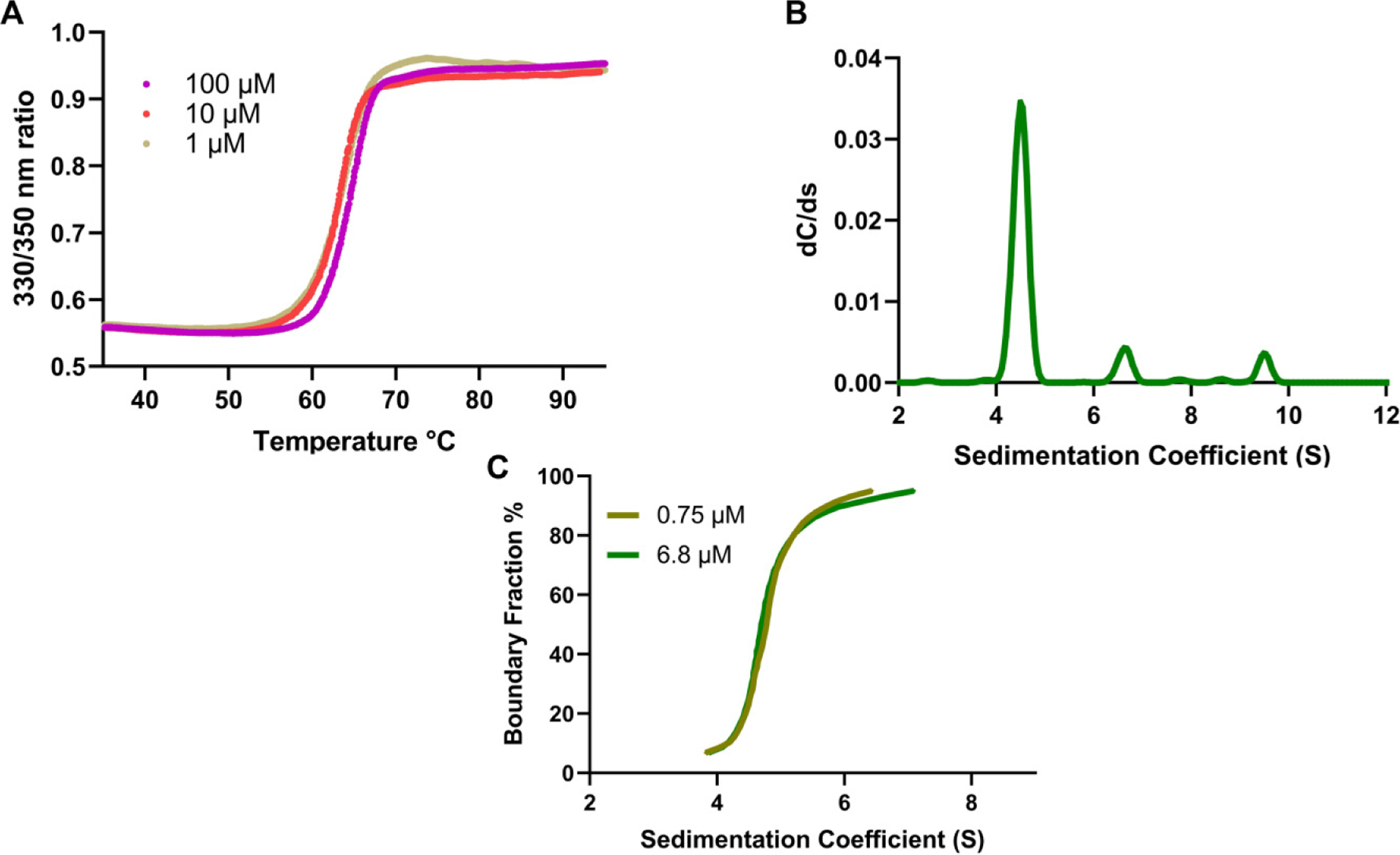
Melting curve and sedimentation velocity analysis of iPPase. (A) Tycho NT.6 melting curve analysis of different concentrations of iPPase. (B) Sedimentation coefficient profile corresponding to iPPase dimer, tetramer, and hexamer. (C) Overlay of two enhanced van Holde–Weischet plots of 6.8 and 0.75 μmol/L concentrations suggests concentration-independent oligomerization.

The first orthogonal validation we chose was melting point analysis through *A*_330/350_ ratio measurements. With the Tycho NT.6, a protein can be quickly and easily analyzed by comparing the unfolding profile and structural integrity at different concentrations. Melting point analysis was performed on yeast iPPase at 0.5, 5, and 50 μmol/L (Fig. 3A). Each melting curve performed at different concentrations shows the same pattern, with melting temperatures for 0.5, 5, and 50 μmol/L of 64.0 ± 0.1, 63.9 ± 0.1, and 64.8 ± 0.1 °C, respectively. Additionally, we chose AUC as an orthogonal validation to confirm our experimental observations of oligomeric assembly. One of the advantages of using AUC is that biomolecules can be characterized at much lower concentrations than many other techniques such as SEC–MALS–DLS. As presented in Fig. 3A, AUC analysis of affinity-purified (Fig. 1B) carried out at 6.8 μmol/L suggested the presence of three distinct peaks for iPPase in solution (Fig. 3B). The sedimentation coefficient for the most prominent species is 4.5 S, followed by 6.5 S and 9.5 S for the tetramer and hexamer, respectively. Additionally, an enhanced van Holde–Weischet (VHW) Demeler and van Holde 2004) transformation was performed (Fig. 3C) on two AUC runs at different concentrations (6.8 μmol/L compared to 0.75 μmol/L). These runs resulted in nearly identical VHW plots with most of the concentration around the 4.5 S value range, consistent with the dimer peak, and smaller concentrations extending above 6 S, consistent with the tetrameric and hexameric species. These results confirm that yeast iPPase can adopt quaternary arrangements in solution. These quaternary arrangements are of unknown enzymatic activity and may, or may not, differ from the enzymatically active dimer.

### Low-resolution structural characterization of yeast iPPase

**Fig. 4.**
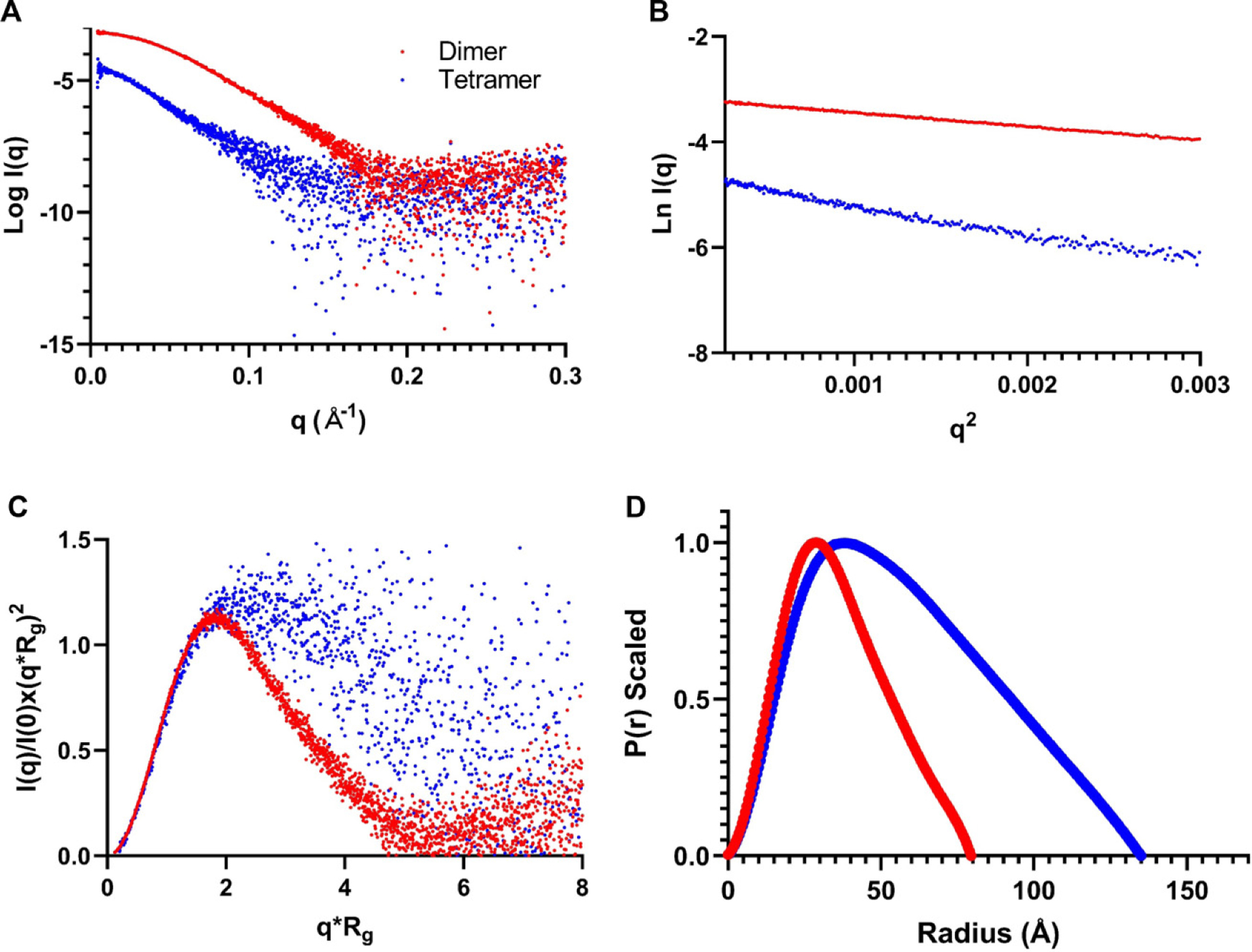
Characterization of iPPase oligomerization via SAXS. (A) Scattering intensity (log *I*(*q*)) vs. scattering angle (*q* = 4*π* sin *θ*/*λ*) represents merged SAXS data. (B) Guinier analysis (ln(*I*(*q*)) vs. *q*2) allows for homogeneity interpretation and determination of *R*_g_ via the low-angle region data. (C) Dimensionless Kratky plots (*I*(*q*)/*I*(0) × (*qR*g)^2^ vs. *qR*g) demonstrate that iPPase folds into a relatively globular state(s). (D) Pair-distance distribution (*P*(*r*)) plots for both iPPase peaks represent their maximal particle dimensions and allow *R*_g_ determination from the entire SAXS dataset.

Low-resolution structural information for iPPase was studied using affinity iPPase. First, the sample was injected into an HPLC instrument connected with the SAXS device to remove any aggregates. SAXS data from the prominent and tetrameric peaks were buffer subtracted, merged, and presented in Fig. 4A. It should be noted that there was not enough homogenous scattering intensity to proceed forward with processing the hexameric peak. The merged data were processed using the Guinier method (plot of *I*(*q*) vs. *q*^2^), which allows for analysis of purity and determination of the *R*_g_ from the low-*q* region data (Guinier et al. 1956). Figure 4B represents the Guinier plots for the iPPase dimer and tetramer, with the low-*q* data linearity demonstrating that both populations are monodispersed and are free of aggregation. Guinier analysis resulted in *R*_g_ values of 28.16 ± 0.04 and 45.77 ± 0.41 Å for iPPase dimer and tetramer, respectively (Table 2). Once the monodispersity of each peak was confirmed, the SAXS scattering data from Fig. 4A was further processed to obtain dimensionless Kratky plots allowing for the detection of the relative foldedness of each population (Patel et al. 2017; Pérez and Vachette 2017). The dimensionless Kratky plot for the dimeric state demonstrates that it is well folded and globular (Durand et al. 2010) in solution with a maximal *y*-axis value of 1.15 (*I*(*q*)/*I*(0) × (*qR*_g_)^2^) (Fig. 4C). The tetrameric state demonstrates a *y*-axis maxima value of 1.5.

Next, the one-dimensional data from Fig. 3A were transformed into the real-space electron pair-distance distribution function (*P*(*r*)) (Fig. 4D). Using the *P*(*r*) plot, *R*_g_ and *D*_max_ for both oligomeric states were calculated. *P*(*r*) analysis resulted in *D*_max_ values of 80 and 135 Å for the iP Pase dimer and tetramer, respectively, and 28.03 ± 0.02 and 45.70 ± 0.15 Å for the *R*_g_ values for the iPPase dimer and tetramer, respectively. These *R*_g_ values are highly similar to the ones provided by the Guinier analysis, indicating an excellent fit of the data. Furthermore, the shape of the *P*(*r*) plot can indicate the solution conformation, with the iPPase dimer displaying an expected Gaussian shape, typical for globular proteins. The iPPase tetramer displays a right-side skewed Gaussian distribution, indicating an extended conformation.

**Fig. 5.**
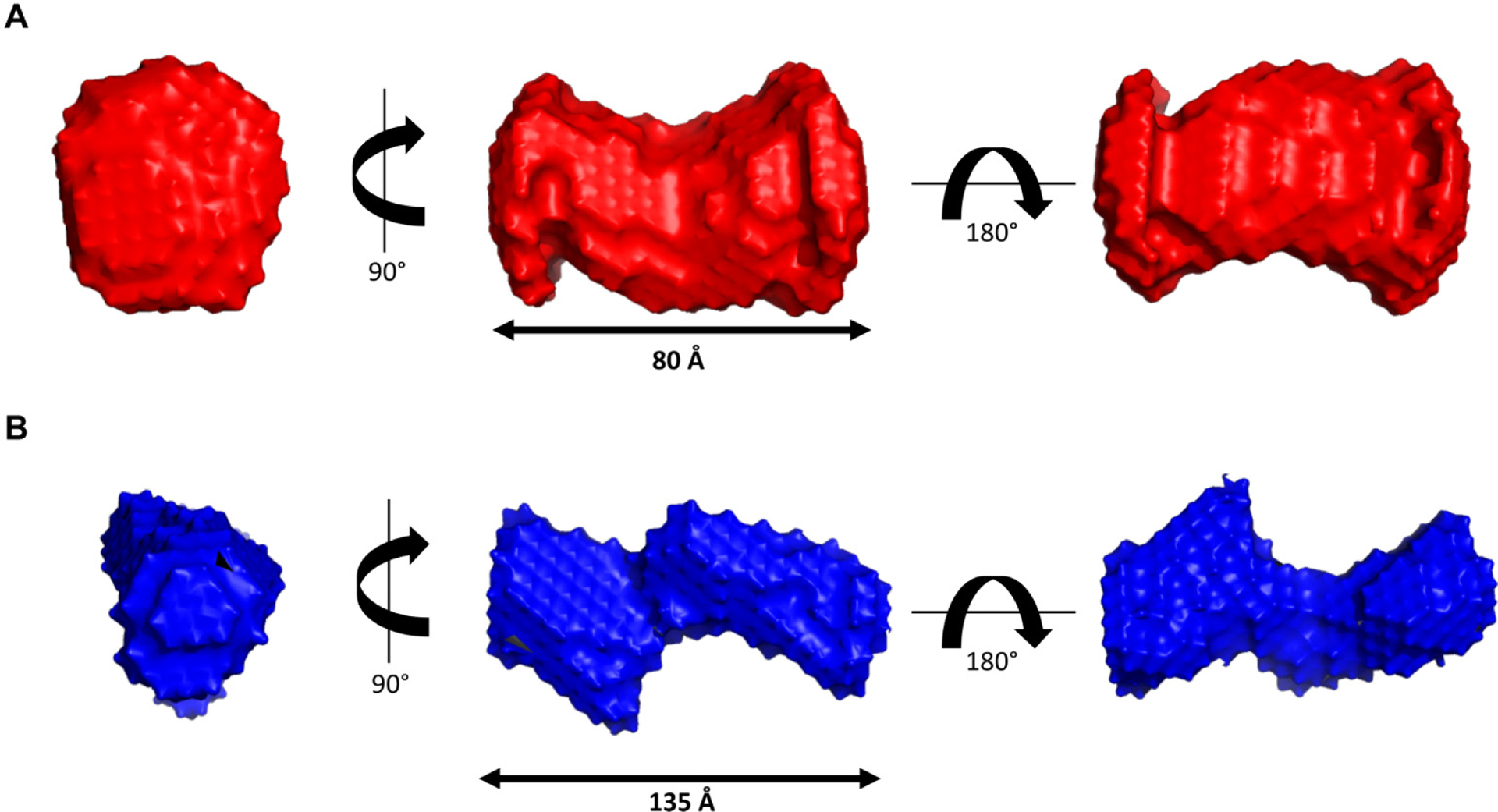
Low-resolution structural representations of iPPase oligomerization. (A) Three structures representing a 90° rotation about the *y*-axis and a 180° rotation about the *x*-axis from the middle representation of the iPPase dimer. (B) Three structures representing a 90° rotation about the *y*-axis and a 180° rotation about the *x*-axis from the middle representation of the iPPase tetramer.

DAMMIN was utilized to generate low-resolution 3D structures of the iPPase dimer and tetrameric oligomerization states. We calculated 12 models for both the iPPase dimer and tetramer with a good agreement with the experimental scattering data and calculated scattering data. The *χ*^2^ values for both cases were ~1.1, representing an agreement between the experimentally collected and low-resolution model-derived scattering data (Table 2). Next, DAMAVER was used to rotate and align all models, obtaining an averaged filtered structure for each oligomeric state (Fig. 5). For each state, the goodness of the superimposition of individual models was estimated by the overlap function; normalized spatial discrepancy (NSD). The NSD value for the model agreement of the iPPase dimer was 0.627 and 0.881 for the tetramer, suggesting a good agreement between the models of each oligomeric state. Figure 5 presents both average filtered structures for the dimeric and tetrameric organization of iPPase and shows different orientations of each structure.

### High-resolution characterization of yeast iPPase tetramerization

**Fig. 6.**
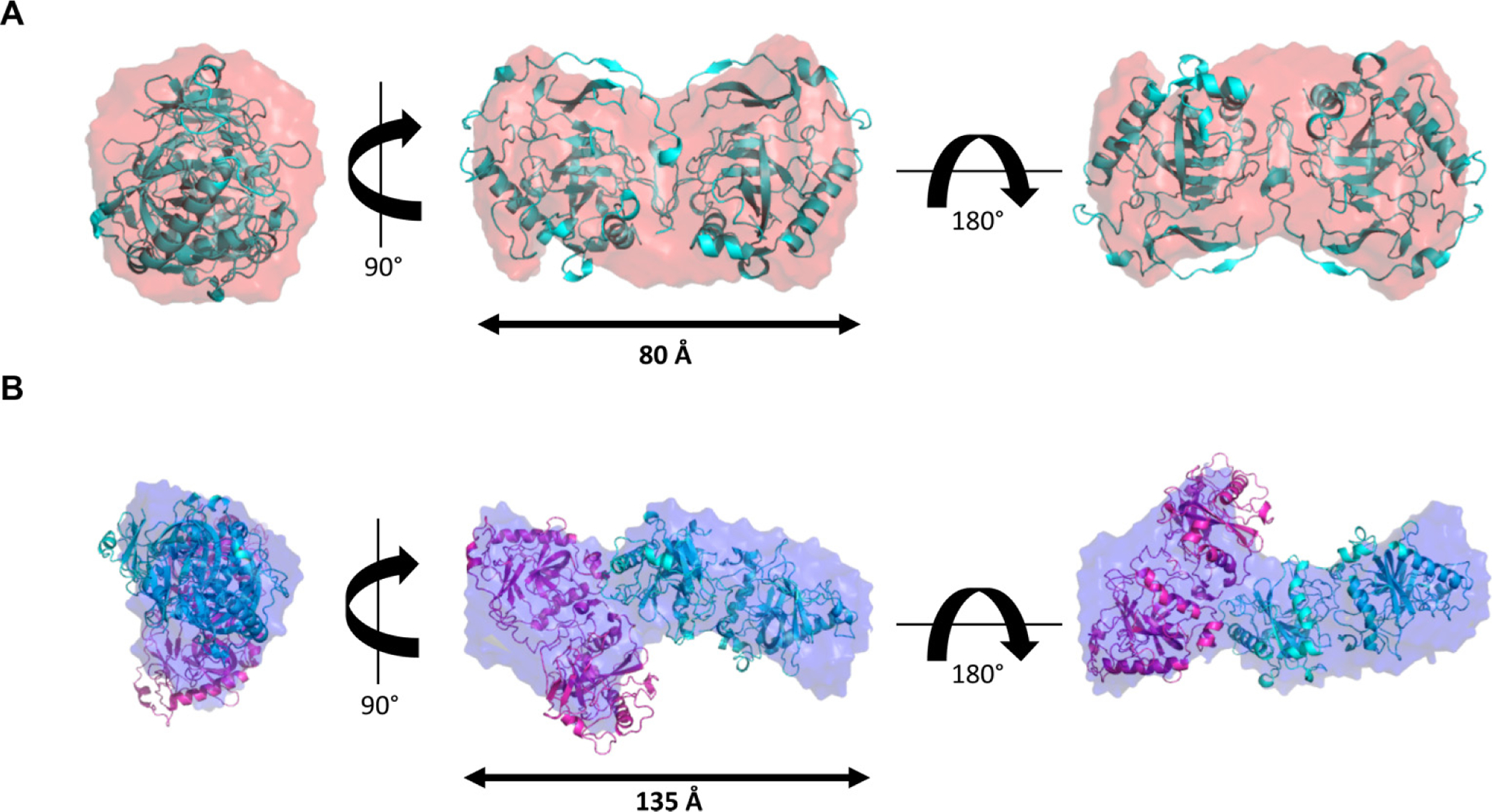
High-resolution representations of the iPPase dimer and tetramer. (A) Three structures representing a 90° rotation about the *y*-axis and a 180° rotation about the *x*-axis from the middle representation of the iPPase dimer with an overlaid high-resolution crystal structure. (B) Three structures representing a 90° rotation about the *y*-axis and a 180° rotation about the *x*-axis from the middle representation of the iPPase tetramer with an overlaid high-resolution crystal structure docked via ClusPro.

After calculating low-resolution structures for iPPase, calculations were performed to fit the high-resolution structure into the generated low-resolution models. DAMSUP was utilized to orient the known crystal structure (PDB ID: 117e) into our low-resolution structure. Visually, the high-resolution structure fits well with our low-resolution structure (Fig. 6A). Next, CLUSPRO was utilized to dock the iPPase dimeric crystal structure into a plausible tetrameric arrangement generating 100 possible conformations. We screened these 100 possible conformations against our raw scattering data, choosing the model with the lowest *χ*^2^ value (1.41), and overlaid it against our low-resolution model (Fig. 6), which indicates that the low-resolution structure of dimeric iPPase can accommodate a high-resolution model of two dimeric subunits of iPPase.

## Discussion

As iPPase is an essential enzyme for IVT reactions, it is paramount that any overexpression and purification will need to result in a large quantity of pure product. Initial affinity purification resulted in a large quantity iPPase (Fig. 1A); however, further confirmation was needed with additional bands being present. The affinity-purified iPPase was subjected to a common additional purification step: SEC. The protein was processed through an S200 SEC column and visualized on an SDS-PAGE. Figure 1B shows three distinct peaks in the SEC chromatogram, and when visualized, all three peaks displayed the same size (Fig. 1C). Peak 3, which we believed to be the dimeric iPPase, still showed a larger protein species similar to affinity purification, which we believed to be a small amount of not fully denatured iPPase dimer.

A test IVT was performed to evaluate if the lab-purified iPPase is active and works well compared to the commercially available iPPase. As presented by the urea-PAGE (Fig. 1D), there was no difference in the amount and quality of RNA produced between the purchased and lab-purified iPPase. Additionally, a lower concentration of lab-made iPPase 0.18 μmol/L was utilized than the commercially purchased iPPase (0.29 μmol/L). This concentration is promising for labs involved with RNA preparation as overall, the lab-made iPPase is just as effective as commercial vendors but a lot cheaper to prepare and purify.

The ambiguity of this additional protein band led us to further biophysical characterization through AUC, SEC–MALS–DLS, and eventually SAXS. First, we chose a combination of MALS and DLS coupled with SEC. MALS will importantly yield the absolute molecular weight across a peak when coupled to SEC, and DLS will gather the radius of hydration from the same peak. Therefore, utilizing SEC–MALS–DLS, the most prominent peak had a molecular weight that matched the dimer of iPPase (63.78 kDa) (Fig. 2A). This result was expected, as it is known that yeast iPPase is active as a dimer (Heinrikson et al. 1973). The middle peak gave an absolute molecular weight very close to a tetramer of iPPase (125 kDa). This was a surprising result because iPPase has not been characterized to exist in tetrameric conformation. Finally, the first eluted peak has a molecular weight very close to what would be the hexamer (186 kDa). This observation is interesting because it has been shown that bacterial iPPase forms an active hexamer (Harutyunyan et al. 1997), but it has never been shown that yeast iPPase can form any other tertiary structures apart from dimers. Next, the SEC–DLS experimental results were analyzed. These results corroborated the SEC–MALS results showing that while each SEC peak showed a single band on SDS-PAGE, corresponding to the monomer molecular weight, they have different hydrodynamic radii (Fig. 2B). The third peak corresponding to the dimeric form had an *R*_H_ of 3.6 nm, consistent with proteins of similar size such as BSA (Jachimska et al. 2008). The second and third peaks, corresponding to the tetramer and hexamer, resulted in *R*_H_ of 5.24 and 6.31 nm, respectively, increasing as molar mass increased. Therefore, based on SEC–MALS–DLS, there is strong evidence that yeast iPPase forms both tetramers and hexamers in solution and the active dimer. However, with the large concentration used in SEC–MALS–DLS, these observations might be artificially induced, so an orthogonal approach was needed, which could be performed at a lower concentration.

The first orthogonal validation we chose was melting point analysis via Tycho NT.6. With the Tycho NT.6, a protein can be quickly and easily analyzed by comparing the unfolding profile and structural integrity at different concentrations (Mohamadi et al. 2017). The Tycho NT.6 does this by comparing the ratio of intrinsic fluorescence at 350 and 330 nm, contributed by tryptophans and tyrosines. Therefore, if iPPase oligomerization was induced by high concentration, there should be a difference in the melting curves between different concentrations. Figure 3A presents the results at three distinct concentrations: 0.5, 5, and 50 μmol/L. The melting curves for 5 and 50 μmol/L present a very similar melting temperature of 64.0 and 63.9 °C, respectively, while 1 μmol/L concentration resulted in 64.8 °C, which is still similar. Therefore, based on the melting temperature evidence, it does not seem that increasing the concentration of iPPase results in oligomerization.

To further verify that iPPase oligomerization is concentration-independent, AUC was chosen. AUC is a powerful technique, whereas biomolecules are subjected to extremely high centrifugal force and separated based on size, anisotropy, and density (Patel et al. 2016). AUC requires a relatively low concentration of the target sample (0.2–0.8 OD) and can be measured off-peak to further lower the target molecular concentration making it an ideal orthogonal method to pair with SEC–MALS–DLS. AUC analysis was performed at 6.8 μmol/L, considerably less than the SEC–MALS analysis, and displayed the presence of three different quaternary structures in the pure iPPase sample. We can infer that the most prominent peak is the dimer as it has the highest concentration and the smallest sedimentation coefficient of 4.5 S. Then, as the species get larger, from tetramer to hexamer, the sedimentation coefficient increases from 6.5 S to 9.5 S. To determine whether the oligomerization was concentration-dependent, AUC experiments were performed at 220 nm, which allowed for much lower concentrations compared to 280 nm (0.75 μmol/L vs. 6.8 μmol/L). Figure 3C presents enhanced VHW plots showing boundary fraction % as a function of sedimentation. If iPPase oligomerization were concentration-dependent, the relative fraction of each state (dimer:tetramer:hexamer) would shift in response, and the plots would look significantly different. Both plots are congruent when overlaid, suggesting that oligomerization is not concentration-dependent within the concentration range tested. With melting curve analysis, SEC–MALS–DLS, and AUC, this evidence suggests that the formation of different iPPase oligomeric structures is not dependent on concentration within the extensive range tested.

Next, we utilized SEC–SAXS to determine the low-resolution structure of iPPase in solution for multiple reasons. First, a structural method was needed that could simultaneously separate biological molecules by size and allow the determination of structural features. Second, we needed a method in which an upper concentration limit would not be problematic. Concentration consideration was vital because it was determined that the molar ratio between the oligomeric states (dimer:tetramer:hexamer) is 55:3:1. Therefore, the amount of purified iPPase sample required to generate SAXS data would need to be enormous to ensure that the tetramer and the hexamer data would be usable. Unfortunately, while our injection of 450 μmol/L was enough to get a large signal-to-noise ratio for dimeric and tetrameric peaks, we did not obtain a sufficiently high signal-to-noise ratio for the hexametric peak. This result is most likely due to the amount of protein loaded (13.28 mg/mL) above the SEC column recommended 10 mg/mL, which would broaden any peaks. Ultimately, we continued evaluating the dimer and tetrameric peaks, even though a usable hexameric peak was unattainable.

Initial SAXS analysis shows the difference in the concentration between the tetrameric and dimeric states by comparing how much tighter the data groups together as *q* increases in the dimer versus the tetramer (Fig. 4A). The Guiner plots also mirror this notion, with the low-*q* region of the dimer Guiner analysis showing virtually no data spread compared to the tetramer. Additionally, the Guiner analysis resulted in what we expected; the tetrameric oligomerization has a considerable increase in *R*_g_ (28.16Å vs. 45.77 Å) (Fig. 4B). This increase suggests that the oligomeric interface between the two stable dimers is likely an end-to-end interaction. This observation is further reinforced by comparing the dimensionless Kratky plots of both oligomeric states. The dimeric dimensionless Kratky has a *y*-maxima of ~1.1, typical of globular proteins in solution. Comparatively, the tetramer has a dimensionless Kratky *y*-maxima of ~1.5 and far less of a Gaussian-like distribution (Fig. 4C). This distribution is evidence of a more extended biomolecule, similar to nucleic acids (Leggio et al. 2008; Kikhney and Svergun 2015; Mrozowich et al. 2020; Nelson et al. 2021). Comparing the *D*_max_ values for the dimer and tetramer (80Å vs. 135 Å) reveals that the oligomeric interface is not perfectly end-to-end; if this were the case, the *D*_max_ would double (Fig. 4D). However, since the *D*_max_ increases considerably, the oligomeric interface is likely an end-to-end interaction, not lengthwise. From the paired-distance distribution analysis of both oligomeric states, we observed that the real-space *R*_g_ values match almost identically with the reciprocal space *R*_g_ values derived from Guiner analysis for both the dimer and tetramer, respectively (28.16Å vs. 28.93 Å and 45.77Å vs. 45.70 Å). The similarity in *R*_g_ values suggests that both datasets are in excellent agreement and worthy of proceeding to 3D modeling.

3D ab inito bead modeling was performed via DAMMIN, resulting in low-resolution structures for both dimeric and tetrameric iPPases. Figure 5 represents both the dimer and tetramer of iPPase in different orientations, showing that, as the previous data suggested, the tetramer seems to consist of two dimers in an extended conformation. The representative structures for dimeric and tetrameric iPPase oligomeric states studied have a *χ*^2^ value of 1.1, suggesting that the models have a good fit to the raw scattering data. Both dimer and tetramer are representative of a filtered average of 12 models with an NSD of 0.627 and 0.881, respectively. These NSD values show that the filtered models all have a very good fit to each other, and the representative model shown is an accurate representation of the model in solution.

Furthermore, since the high-resolution structure of yeast iPPase has been previously determined (Heikinheimo et al. 1996; Tuominen et al. 1998; Kuranova et al. 2003), we compared the high-resolution structure with our low-resolution SAXS models. As presented in Fig. 6A, the high-resolution structure fits well into our solution scattering envelope, clearly showing both monomeric units of the crystalized homodimer. Overlaying high-resolution information into our SAXS envelope was an essential final step in validating our previous biophysical SEC–MALS and DLS data. Subsequently, we utilized CLUSPRO to dock the dimeric high-resolution crystal structure of iPPase to obtain 100 possible conformations of the iPPase tetramer. The docked models were screened using SAXS data and CRYSOL to determine a conformation that may represent a tetrameric oligomerization state. We chose the model for a tetramer that agreed the most with the SAXS data, as evaluated by the lowest *χ*^2^ value, and represented it in Fig. 6B. The high-resolution docked model fits very well visually with our SAXS envelope, validating our previous biophysical characterization that iPPase makes higher order oligomeric species in addition to its biologically active homodimer.

Finally, we calculated the total cost of IVT reactions using a commercially purchased kit. One commonly used kit is the Ampliscribe^™^ T7-Flash^™^ Transcription Kit (Lucigen, Middleton, WI, USA) which sells for on average USD 241.00 for 25 reactions. If we scale this up to a 1 mL IVT reaction, the approximate cost would be USD 482.00. Alternatively, when reagents are purchased separately, which include ATP, UTP, GTP, CTP (Sigma–Aldrich, Oakville, ON, Canada), GMP (Sigma–Aldrich, Oakville, ON, Canada), RiboLock (Thermo Fisher Scientific, High River, AB, Canada), in combination with in-house T7 polymerase and affinity-purified iPPase, the cost for 1 mL IVT reaction would be approximately USD 30.00. We calculated the cost of producing the iPPase to be relatively negligible (media cost is ~$5.00/L), aside from the up-front cost of reusable nickel affinity resin. Thus, the laboratory-produced iPPase provides a cost-effective solution for large-scale IVTs. Finally, since it was determined that all three contaminant peaks in affinity-purified iPPase were oligomeric species of iPPase and that affinity-purified iPPase produced similar in vitro transcribed RNA, we believe that affinity purification alone is sufficient when using iPPase for IVT reactions.

Ultimately, this work provides a multi-faceted biophysical approach to characterizing the multiple oligomeric states of yeast iPPase. Furthermore, the expression and purification pipeline can provide a large quantity of active yeast iPPase to help offset high research costs incurred during RNA research. Ideally, a reduction in RNA research costs can significantly affect the ability of research labs to produce usable quantities of RNA, reducing the barriers toward RNA research. Moreover, the preparation of RNA therapeutics often requires immense amounts of RNA material, especially RNA-based vaccine development such as the recent COVID-19 vaccines. Therefore, it is imperative that production costs be reduced as much as possible to alleviate the overall costs of these RNA therapeutics.

## Figures and Tables

**Table 1.** Components and final concentrations of the in vitro transcription reaction.

Component	Final concentration
5X TRAB	1 ×
DTT	10 mmol/L
NTPS	3 mmol/L
GMP	5 mmol/L
iPPase	0.29 μmol/L (purchased)/0.18 μmol/L (lab)
T7 polymerase*	0.18 μmol/L
Ribolock	0.5% (*v*/*v*)
Plasmid DNA	18 ng/*μ*L
MilliQ	Up to desired volume

* T7 polymerase produced in-house.

**Table 2.** Small-angle X-ray scattering parameters.

	Dimer	Tetramer
Sequence molecular weight (kDa)	64.6	129.2
Guiner, *R*_g_ (Å)	28.16 ± 0.04	45.77 ± 0.41
Kratky maxima	1.15	1.5
*P*(*r*) *R*g (Å)	28.03 ± 0.02	45.70 ± 0.15
*D*_max_ (Å)	80	135
*x* ^2^	1.14	1.09
NSD	0.627	0.881
